# Atrioventricular Cross-Talk Leading to Ventricular Pacing Inhibition in a Dual-Chamber ICD

**DOI:** 10.1155/2011/624508

**Published:** 2011-08-22

**Authors:** Luuk Debie, Bernard Broers, Jurren van Opstal, Berry M. van Gelder

**Affiliations:** ^1^Department of Cardiology, University Hospital Maastricht, 6202 AZ Maastricht, The Netherlands; ^2^Department of Cardiology, Catharina Hospital, Michelangelolaan 2, 5623 EJ Eindhoven, The Netherlands

## Abstract

We report atrio-ventricular cross talk in a patient with a dual chamber ICD leading to ventricular pacing inhibition. This was observed in an ICD without the ventricular safety pace option, which normally is a sufficient protection against this phenomenon. Cross talk could be prevented effectively by reprogramming ventricular sensitivity to a less sensitive setting.

## 1. Introduction

Atrioventricular (A-V) cross-talk resulting in ventricular inhibition is a known phenomenon since the introduction of devices with dual-chamber pacing and ventricular sensing capabilities [1]. Cross-talk sensing resulting in ventricular inhibition is a rare phenomenon in modern ICD's and pacemakers. Bipolar electrodes, sophisticated sensing and pacing circuits, and introduction of programmable blanking periods have reduced the susceptibility for AV cross-talk. If cross-talk occurs, inhibition of ventricular pacing can be prevented by the ventricular safety pace option. We report our experience in a modern ICD without the latter option. 

## 2. Case Report

An 83-year-old male patient had a DDD pacemaker (Medtronic Kappa 733) implanted in 1995, because of complete atrioventricular (AV) block. Due to progressive heart failure, the pacemaker was upgraded in 2004 to an ICD with cardiac resynchronization therapy (Guidant Contak Renewal M179, CRT-D). For this purpose a Guidant 0138 ICD shock lead was implanted in the right ventricle and a Medtronic Attain 4194 in a posterolateral branch of the coronary sinus for left ventricular pacing. The preexisting Medtronic 4524 atrial lead was used for atrial pacing and sensing, and the bipolar right ventricular lead was abandoned. 

**Figure 1:**
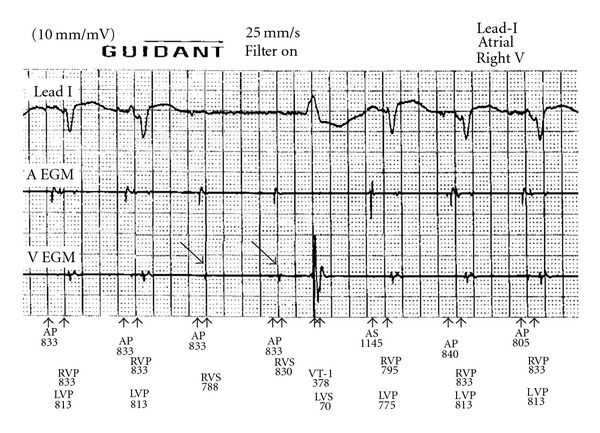
Pacing at DDD 65/110 beats per minute. Shown from top to bottom are the lead I, atrial electrogram (A egm), right ventricular electrogram (V egm), and annotations. AP: atrial pacing; AS: atrial sensing; RVP: right ventricular pacing; RVS: right ventricular sensing; LVP: left ventricular pacing. Cross-talk inhibition occurs at the fourth and fifth complex (arrows).

A hospital admission because of ventricular tachycardias showed successful termination of these tachycardias by ICD shock therapy. Except shock therapy, the patient complained of dizzy spells, which at that time were attributed only to the ventricular tachycardias. However, at the time of device interrogation, the ECG showed intermittent ventricular inhibition.  Figure 1 demonstrates intermittent ventricular sensing followed by inhibition after atrial pacing. The interval between atrial pacing and ventricular sensing was estimated from 60 to 70 ms in this recording. Practically sensing is anticipated immediately after time out of the ventricular blanking period, which was programmed at 65 ms [2]. 

**Figure 2:**
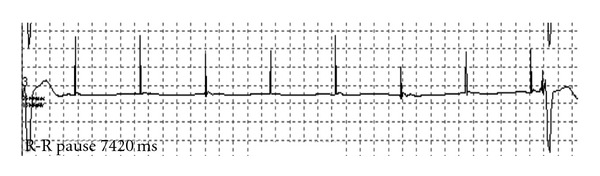
Holter recording showing only atrial pacing spikes without ventricular response resulting in a pause of 7.4 seconds.

Inhibition did not occur during atrial sensing, which is a strong indicator of cross-talk sensing. A Holter recording performed 24 hours prior to the device checkup revealed several periods of asystole with duration up to 7.4 seconds as a consequence of cross-talk sensing (Figure 2). The chest X-ray showed normal lead positions; there was no evidence of lead damage which was confirmed by unchanged values of lead impedances.

The pacing parameters were DDD 65 till 110 beats/min and dynamic AV delay 160 ms/100 ms pacing output in atrial and RV 2.6 V at 0.06 ms, and LV 3.0 V at 0.5 ms, the RV-blank after A-pace was 65 ms, and the RV sensitivity was set to nominal.

**Figure 3:**
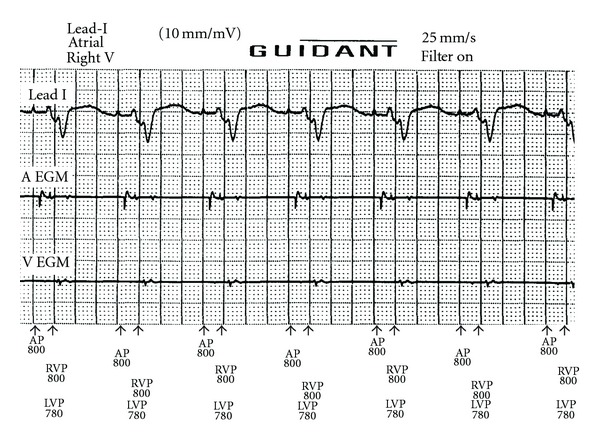
Restoration of normal function without cross-talk sensing after reduction of the ventricular sensitivity to the least sensitive setting. Abbreviations are similar to Figure 1.

Neither prolongation of the ventricular blanking period to 85 ms nor decreasing atrial output to 1.0 V at 0.5 ms could eliminate cross-talk sensing [3]. Finally, cross-talk sensing disappeared after programming right-ventricular sensitivity from nominal to the least sensitive setting (Figure 3). Proper sensing during intrinsic rhythm and ventricular fibrillation during DFT testing was confirmed at this sensitivity setting.

## 3. Discussion

Cross-talk sensing is a rare phenomenon, which can be life-threatening or symptomatic if it results in ventricular inhibition, as illustrated in this case. In patients with complete AV block or in absence of underlying rhythm, extra attention is warranted. 

There are several factors promoting cross-talk sensing. Increase of atrial output (amplitude and pulse width), more sensitive setting of the ventricular channel, unipolar electrode configuration of the atrial and ventricular channel, and increasing pacing rates are known causes of cross-talk sensing. Exceptional is cross-talk sensing by increase of ventricular output [4]. 

When cross-talk sensing cannot be eliminated by adaptation of one or more of the here a forementioned parameters or by lengthening of the ventricular blanking period, ventricular inhibition can be prevented by a safety feature called “Ventricular Safety Pacing (VSP).” This method uses a brief sensing period (crosstalk sensing window) after the ventricular blanking period starting at the atrial stimulus [5]. Any electrical ventricular event sensed during this window is regarded to be cross-talk. Ventricular sensing during this window results in ventricular pacing at the end of the safety window, which can be recognized from the ECG by shortening of the AV interval [6, 7]. However, also other sense events occurring in the cross-talk sensing window can cause a safety ventricular output pulse. Examples are premature ventricular beats, premature junctional beats, and normally conducted ventricular events that occur after atrial undersensing. Therefore, safety output pulses are delivered at a relative short AV interval, thus preventing stimulation in the early repolarization phase of the ventricle. However, when the premature or intrinsic beat occurs during the ventricular blanking period the programmed AV interval will be timed out and a ventricular output is delivered at the end of this AV interval. Therefore, the use of a long AV interval should be avoided, and relatively short AV intervals are advised (typically, between 100 and 120 ms) [8]. 

Unfortunately the current device was not equipped with the feature of VSP, and ventricular inhibition could only be prevented by reduction of the ventricular sensitivity. The latter option is not the first choice in patients with ICDs, because reduction of sensitivity may result in undersensing during ventricular arrhythmias.
